# Anxiety Symptoms Are Associated With Higher Psychological Stress, Poor Sleep, and Inadequate Sleep Hygiene in Collegiate Young Adults—A Cross-Sectional Study

**DOI:** 10.3389/fpsyt.2021.677136

**Published:** 2021-07-01

**Authors:** Md Dilshad Manzar, Ahmad H. Alghadir, Masood Khan, Mohammed Salahuddin, Abdulrhman Albougami, Jestoni D. Maniago, Brian A. Vasquez, Seithikurippu R. Pandi-Perumal, Ahmed S. Bahammam

**Affiliations:** ^1^Department of Nursing, College of Applied Medical Sciences, Majmaah University, Al Majmaah, Saudi Arabia; ^2^Rehabilitation Research Chair, College of Applied Medical Sciences, King Saud University, Riyadh, Saudi Arabia; ^3^Department of Pharmacy, College of Medicine and Health Sciences, Mizan-Tepi University (Mizan Campus), Mizan-Aman, Ethiopia; ^4^Pharmacology Division, Department of BioMolecular Sciences, University of Mississippi, Oxford, MS, United States; ^5^Somnogen Canada Inc., Toronto, ON, Canada; ^6^The University Sleep Disorders Center, College of Medicine, King Saud University, Riyadh, Saudi Arabia; ^7^National Plan for Science and Technology, College of Medicine, King Saud University, Riyadh, Saudi Arabia

**Keywords:** anxiety, perceived stress, sleep quality, sleep hygiene, Leeds sleep evaluation questionnaire

## Abstract

**Background:** Anxiety symptoms, stress, poor sleep, and inadequate sleep hygiene are common in university students and these affect their learning and increase attrition. However, limited knowledge exists about the inter-relationship between these factors among university students in low-middle income countries. Therefore, this study aimed to investigate the prevalence of anxiety symptoms and their relationship with sleep quality, sleep hygiene practices, and psychological stress.

**Materials and Methods:** A cross-sectional study was conducted with a randomly selected sample of students in Mizan-Aman, Ethiopia. Participants completed a self-administered questionnaire, which included questions about socio-demographics, socio-economic factors, the Leeds Sleep Evaluation Questionnaire-Mizan, Sleep hygiene index, Perceived stress scale-10 (PSS-10), and Generalized anxiety disorder-7 scale (GAD-7).

**Results:** The prevalence of anxiety symptoms was about 22%. Multivariate regression analysis showed that both anxiety status χ^2^[(13, *N* = 480) = 82.68, *p* < 0.001], and increasing levels of anxiety (model adjusted R2 = 0.204, *p* < 0.001) were associated with greater psychological stress, inadequate sleep hygiene practices, and poor sleep quality scores after adjusting for age, gender, attendance, substance use, years of university education, time spent in athletic activity every day, and frequency of tea/coffee consumption.

**Conclusion:** There was a high prevalence of anxiety symptoms in this study population, and this condition was associated with psychological stress, poor sleep, and inadequate sleep hygiene parameters. These results suggest a need to address the various aspects of mental health and its diverse sleep correlates in university students.

## Introduction

Anxiety is one of the common mental health disorders (global prevalence of 3.7%), associated with significant comorbidity and mortality (1). Anxiety is often comorbid with depression or other anxiety disorders, which renders its diagnosis and treatment complicated (2). As a result, anxiety remains undiagnosed and undertreated in most primary care settings (3). Anxiety has added indirect costs to the economic burden of countries owing to significant loss of personnel productivity and increased use of primary health care services (4). As such, it is important to identify anxiety for early diagnosis and treatment. This is more relevant for university students of the present-day world as mental health issues are common and usually associated with pressure to achieve higher academic grades, a transition to a higher level of studies, and pressure to succeed (5). Estimates vary but a large proportion of college-going students show stress (~51–67%), and anxiety symptoms (~34–56%) (6, 7).

Poor sleep quality adversely influences day-to-day activities and may predispose affected individuals to poor health and quality of life outcomes and mental health issues such as anxiety, depression, and other problems (8). There are pieces of evidence showing that anxiety is correlated with poor sleep quality in insomnia (9, 10). Inadequate sleep hygiene practices are correlated with measures of anxiety and stress in Saudi university students (11). However, the association was determined using bivariate analysis, and therefore it is desirable to further investigate this relationship in other populations using multivariate analysis. Sleep quality is associated with psychological distress and inadequate sleep hygiene among Iranian pre-clinical students (12). We recently found that insomnia is highly prevalent among university students and that it is associated with anxiety, and inadequate sleep hygiene practices (13). However, to the best of our knowledge, no study investigated anxiety, and its association with psychological stress, sleep quality and sleep hygiene.

Moreover, two recent studies in the Ethiopian population determined the prevalence and determinants of anxiety in healthcare workers (14) and women attending prenatal care (15). However, there are few reports which determined the prevalence of anxiety in the Ethiopian student population and studied its associated conditions. Therefore, this study was planned to investigate the prevalence of anxiety and its relationship with psychological stress, sleep quality, and sleep hygiene practices among Ethiopian university students.

## Materials and Methods

### Study Design and Participants

A total of 17936 students were enrolled in both the campuses located at Mizan city and Tepi city of the Mizan-Tepi University (MTU) in regular and continuous programs in 2017–18 academic year. Haile et al. had reported a prevalence of 63.1% for common mental disorders especially anxiety and depressive symptoms in a similar setting of university students in Ethiopia (16). Therefore, a sample size of 598 was estimated using 5% as margin of error, confidence interval of 99%, population (all students of MTU), and 63.1% as expected response distribution. We randomly selected the Mizan campus for the recruitment of participants in this cross-sectional study. A list of 750 students was initially prepared by a simple random method. Of these students, 189 declined to participate or were absent on the day of the interview, 36 were excluded after screening for the exclusion criteria, and 26 responses were deleted because of person-level missing values, that is, missing responses for most of the items of the four standard questionnaires. Finally, analysis of a dataset of collegiate young adults (*n* = 499, age: 21.34 ± 2.76 years) is presented. The final sample size was deemed adequate because it was still more than a sample size (351) estimated using 5% as margin of error, confidence interval of 95%, population (all students of MTU), and 63.1% as expected response distribution. Respondents were asked to complete a self-administered questionnaire during in-person meetings with the research team. These meetings were scheduled during the semesters to avoid bias from the examination-related stress. Students who were pursuing higher education from Mizan-Tepi University, Mizan-Aman, Ethiopia were included in the study. Those with an idiosyncratic account of self-reported recollected complaints and those taking neuropsychiatric medications were excluded from the study. Institutional review committee approval from the College of Health Sciences, Mizan-Tepi University, Ethiopia was acquired. Before the collection of data, a precis of the intent and methodology to be implemented was explained to the respondents. The nature of participation was clarified to the respondents as voluntary and their right to terminate participation at any time-point was emphasized. There were no direct benefits for respondents and no risks or chances of harm. It was also communicated to the respondents that they will not receive any fee in exchange for participation. Stringent provisions for privacy and confidentiality were implemented. The study strictly conforms with the Helsinki declaration. Informed written consent for participation and publication were obtained. The study utilized five tools: (i) Generalized anxiety disorder-7 scale (GAD-7), (ii) Leeds Sleep Evaluation Questionnaire-Mizan, (iii) Perceived stress scale-10 (PSS-10), (iv) Sleep hygiene index, and (v) a semi-structured socio-demographics tool. Since the English language is the medium of instruction in all federal universities in Ethiopia, all the tools employed in this study were in English.

### Measures

#### Generalized Anxiety Disorder − 7 Scale

GAD-7 scale is a succinct but scrupulously validated inventory to examine anxiety symptoms (17–19). It has been shown to have sufficient diagnostic validity for vetting a wide-array of anxiety-related disorders: “GAD, panic disorder, social phobia, and post-traumatic stress disorder” (18). A cut-point of 10 for GAD-7 is observed in this study to determine anxiety symptoms. A sensitivity of 68% and specificity of 88% were reported by Kroenke et al. at this cut-point. Seven items are scored using an ordinal scale (0–3) to account for the cumulative regularity of anxiety symptoms within the last 7 days (18). The GAD-7 total score, ranging from 0–21, is equal to the linear summation of all the 7-items (17, 18). The severity of the anxiety increases with the increment in the GAD-7 total score (17, 18). The GAD-7 is a valid instrument for screening anxiety in university students in Afro-Asian countries (19, 20).

#### Sleep Hygiene Index

SHI is a validated 13-item self-report measure of sleep hygiene (21). This study adapted the version with a slightly modified scoring system (dichotomized rating for each of the items: 0 = No; 1 = Yes). SHI total score is the summation of all the items ranging from 0–13, where a lower score infers good sleep hygiene practices and a higher score suggests the otherwise (21). The reliability of this version (dichotomized scoring) is sufficient as indicated by a GLB = 0.85 and Standardized α = 0.66 (22). SHI is valid among university students (11).

#### Leeds Sleep Evaluation Questionnaire-Mizan

Manzar et al. (23) validated the modified version of the LSEQ (i.e., LSEQ-M) (24). The revision was composed to gauge the sleep complaints among the student populace. A 100 mm visual analog scale (VAS) was utilized to rate each of the 10-items (23, 25). Lower scores imply more severe sleep complaints (23). The scores (0–100 range) of each item are transformed into 0–10 range which when summed will yield an LSEQ-M total score ranging from 0–100 (23). LSEQ-M has requisite reliability, adequate internal homogeneity, construct validity, and structural validity in university students (23, 25).

#### Perceived Stress Scale-10

PSS-10 is a succinct (10-items) but scrupulously validated scale of psychological stress (26). PSS-10 is a self-report that appraises the stress experienced by the respondents throughout the month preceding the test. Each item is rated using a 4-point ordinal scale (0 = Never; 4 = Very Often), where higher scores would infer a cumulative regularity of stress-related complaints. The linear summation of all the 10-items will generate the total PSS-10 score (26). The higher the psychological stress level, the higher the PSS-10 total scores. Manzar et al. (27) reported that PSS-10 has adequate psychometric validity when utilized in assessing the psychological stress among Ethiopian university students.

#### Socio-Demographic Questionnaire

A concise questionnaire was utilized to register the socio-demographic characteristics of the respondents (e.g., age, gender, years of education, attendance (% of classes attended), substance use, daily athletic activity duration, and frequency of daily tea/coffee consumption). Substance use item recorded self-reported habitual consumption of alcohol and/or Khat and/or smoking.

### Statistical Analysis

In this study, statistical analysis was performed using SPSS version 26.0. Participants' characteristics were presented using these: mean ± SD (continuous) and percentage and frequency (categorical variables). Chi-square test or Fisher's exact test (categorical variables) and Student's independent *t*-test (continuous variables) were employed for bivariate analysis.

Multivariate analysis was performed using binary logistic and multiple linear regression. Nineteen multivariate outliers [Mahalanobis distance criteria; *X*^2^ (9) = 27.88, *p* < 0.001] and high leverage points and highly influential points (Cook's distance < 1.0) were removed for performing multivariate analysis. There were few univariate outliers in the attendance and age variables but these were not removed because those values were found to be correct and not arising out of data entry mistakes. Other assumptions for binary logistic regression like (i) independence of observations, (ii) absence of multicollinearity among independent variables as determined by the Spearman's correlation coefficients, and (iii) linear relationship with log odds of all independent variables were satisfied by the dataset. Additional assumptions for the multiple linear regression: (i) linear relation between outcome and independent variable as determined by scatterplot and partial regression plots, (ii) homoscedasticity as determined by studentized residuals plotted against the unstandardized predicted values, and (iii) normal distribution of the residual errors were satisfied by the dataset.

All the covariates and independent variables except gender, attendance, years of education, and frequency of daily tea/coffee consumption were significantly correlated with anxiety. However, gender, attendance, years of education, and frequency of daily tea/coffee consumption were used as covariates or predictors because earlier works have shown them to be associated with anxiety (28–31).

## Results

### Participants' Characteristics

Most of the participants (80.6%) were males (Table 1). Three-fourth of the collegiate young adults (75%) were studying in the first 2 years of university education (Table 1). The average of attendance, PSS-10 total score, LSEQ-M total score, and SHI total score was 95.32 ± 6.87, 18.83 ± 6.43, 60.08 ± 21.19, and 6.44 ± 2.39, respectively (Table 1). The prevalence of anxiety symptoms was 21.6%, and the average GAD-7 total score was 7.2 ± 4.3 in the study sample.

**Table 1:** Participants' characteristics and their relationship (bivariate) with anxiety in Ethiopian collegiate young adults.

**Characteristics**	**Mean ± SD** **/frequency (percentage)**	**Normal (*n* = 391)** **mean ± SD** **/frequency (percentage)**	**Anxiety symptoms (*n* = 108)** **mean ± SD** **/frequency (percentage)**	**Statistics**	***P*-value**
Age (yr)	21.34 ± 2.76	21.55 ± 2.99	20.57 ± 1.47	4.71^a^	<0.001
Gender					
Male	402 (80.6)	318 (81.3)	84 (77.8)	0.68^b^	0.41
Female	97 (19.4)	73 (18.7)	24 (22.2)		
Years of education					
1st	186 (37.3)	150 (38.4)	36 (33.3)	6.35^b^	0.18
2nd	188 (37.7)	140 (35.8)	48 (44.4)		
3rd	56 (11.2)	43 (11.0)	13 (12.0)		
4th	39 (7.8)	30 (7.7)	9 (8.3)		
5th	30 (6.0)	28 (7.2)	2 (1.9)		
Attendance	95.34 ± 6.92	95.32 ± 6.87	95.44 ± 7.13	−0.16^a^	0.88
Substance use^*^				0.45^b^	0.50
No	464 (93.0)	362 (92.6)	102 (94.4)		
Yes	35 (7.0)	29 (7.4)	6 (5.6)		
PSS-10 total score	19.69 ± 6.40	18.83 ± 6.43	22.82 ± 5.24	−6.65^a^	<0.001
LSEQ-M total score	57.88 ± 21.18	60.08 ± 21.19	49.90 ± 19.18	4.51^a^	<0.001
SHI total score	6.67 ± 2.32	6.44 ± 2.39	7.47 ± 1.84	−4.80^a^	<0.001
Daily athletic activity duration	39.53 ± 39.59	42.68 ± 40.85	28.15 ± 32.33	3.89^a^	<0.001
Frequency of tea/coffee consumption	1.15 ± 0.63	1.18 ± 0.63	1.06 ± 0.61	1.81^a^	0.07

* *Substance use (self-reported habitual use of alcohol and/or Khat and/or smoking); Anxiety symptoms was screened by Generalized anxiety disorder-7 scale*.

*SD, standard deviation; GAD-7, generalized anxiety disorder-7 scale; SHI, sleep hygiene index; LSEQ-M, an adapted and validated English version of the Leeds sleep evaluation questionnaire; PSS, perceived stress scale-10*.

*Statistics: Chi-square test/ Fisher's exact test for categorical variables and student's t-test for continuous variables*;

a *: t statistic*;

b *: chi-square statistic value*.

### Bivariate Analysis: The Relationship Between Anxiety and Participants' Characteristics

The bivariate analysis predicted that anxiety was more common in students with lower age (20.57 ± 1.47 vs. 21.55 ± 2.99, *p* < 0.001) and those who spent less time in athletic activities every day (28.15 ± 32.33 vs. 42.68 ± 40.85, *p* < 0.001) (Table 1). Both PSS-10 total score (22.82 ± 5.24 vs. 18.83 ± 6.43, *p* < 0.001) and SHI total score (7.47 ± 1.84 vs. 6.44 ± 2.39, *p* < 0.001) were higher in the anxiety group than normal (Table 1). Similarly, the anxiety group showed lower values for LSEQ-M total score (49.90 ± 19.18 vs. 60.08 ± 21.19, *p* < 0.001), indicating poorer sleep than the normal young adults (Table 1).

### Multivariate Analysis: Binary Logistic Regression-Association of Anxiety Disorder With the Level of Stress, Poor Sleep, and Inadequate Sleep Hygiene

A binary logistic regression was run to predict anxiety; this model was adjusted for age, gender, attendance (percentage of lectures attended), substance use (self-reported habitual use of alcohol and/or Khat and/or smoking), years of university education, time spent in athletic activity every day, and frequency of tea/coffee consumption. The model with three predictors, that is, PSS-10 total score, SHI total score, and LSEQ-M total score were significant in comparison to a model with only intercepts; χ^2^(13, *N* = 480) = 82.68, *p* < 0.001. The model with these predictors explained 24.1% of the variance in the classification of anxiety with an accuracy of 78.8%. Increasing scores of PSS-10 and SHI and the decreasing score of LSEQ-M were associated with anxiety status (Table 2).

**Table 2:** Binary logistic regression: association of anxiety with psychological stress, poor sleep, and inadequate sleep hygiene in Ethiopian collegiate young adults.

**Associated conditions**	**AOR (95 % CI)**	***P*-value**	**COR (95 % CI)**	***P*-value**
LSEQ-M total score	0.97 (0.96–0.99)	<0.001	0.98 (0.97–0.99)	<0.001
SHI total score	1.16 (1.03–1.30)	0.01	1.22 (1.10–1.35)	<0.001
PSS total score	1.11 (1.06–1.16)	<0.001	1.12 (1.07–1.17)	<0.001

*CI, confidence interval; AOR, adjusted odds ratio; COR, crude odds ratio*.

*Adjusted for age, gender, attendance (percentage of lectures attended), Substance use (self-reported habitual use of alcohol and/or Khat and/or smoking) and years of university education, time spent in athletic activity every day, frequency of tea/coffee consumption*.

*Anxiety was screened by Generalized anxiety disorder-7 scale; LSEQ-M, an adapted and validated English version of the Leeds sleep evaluation questionnaire; SHI, sleep hygiene index; PSS: perceived stress scale-10*.

### Multivariate Analysis: Multiple Linear Regression-Association of Anxiety Level With the Level of Stress, Poor Sleep, and Inadequate Sleep Hygiene

Further, a multiple linear regression model assessed the extent to which inadequate sleep hygiene, poor sleep, and psychological stress predict changes in anxiety levels. Increasing severity of the anxiety (implied by increasing GAD-7 total score) was predicted by an increasing level of poor sleep (lower LSEQ-M total score), inadequate sleep hygiene (higher SHI total score), and increasing psychological stress (increasing PSS-10 total score) (model adjusted R2 = 0.204, *p* < 0.001) (Table 3).

**Table 3:** Multiple regression predictors of the anxiety level in Ethiopian collegiate young adults.

**Independent variable**	**Beta coefficient**	**Standard error**	***T*-values**	***P*-values**	**Model unadjusted R2; adjusted R2; *P*-value**
Age	−0.069	0.089	−1.525	0.128	0.219, 0.204, <0.001
Gender	0.001	0.468	0.03	0.976	
Attendance	−0.020	0.027	−0.477	0.633	
Daily athletic activity duration (min)	−0.067	0.005	−1.617	0.106	
Frequency of tea/coffee consumption	−0.097	0.307	−2.353	**0.019**	
SHI total score	0.120	0.084	2.726	**0.007**	
PSS-10 total score	0.307	0.03	7.097	**<0.001**	
LSEQ-M total score	−0.186	0.009	−4.359	**<0.001**	
Years of university education	0.006	0.169	0.134	0.894	
Intercept	8.818^*^	3.376	2.612	0.009	

* *Unstandardized beta coefficient for the intercept, for all other independent variables standardized beta coefficient is shown*.

*GAD-7, generalized anxiety disorder-7 scale; LSEQ-M, an adapted and validated English version of the Leeds sleep evaluation questionnaire; SHI, sleep hygiene index; PSS, perceived stress scale-10. Bold values indicate a significant relationship between an independent variable and the dependent variable*.

## Discussion

In the present study, (i) a high prevalence of anxiety was found in the study population of Ethiopian university students, and (ii) both status of anxiety and an increasing level of anxiety were found to be associated with psychological stress, poor sleep quality, and inadequate sleep hygiene practices. To the best of our knowledge, this is the first study to demonstrate the status of having an anxiety and increasing level of anxiety to be associated with poor sleep, inadequate sleep hygiene practice, and psychological stress. The findings of the present study are strengthened by the fact that all the measures were standard questionnaires that have previously been validated in university students (11, 19, 23, 27). Furthermore, the results are reinforced by the fact that the relationship was significant in the multivariate models after adjusting for many of the known covariates (28–31).

The prevalence of anxiety (~22%) in this study was slightly lower than that reported by a recent systematic review, that is, 24.5% based on a summarized finding of 48 articles from 40 countries (32). Ghrouz et al. (33) found a slightly higher level of anxiety symptoms using the GAD-7 scale (30%) in Indian university students (33). This concordance with the findings of a high prevalence rate of anxiety in Ethiopia and the rest of the world indicates a major global public health problem affecting university attending young adults (32, 33). The anxiety-sleep quality relationship in the present study is supported by previous reports. Poor sleep has been one of the consistent predictors of increased anxiety levels in collegiate young adults (33, 34). Zhang et al. reported that psychological stress mediates the relationship between poor sleep quality and anxiety levels among American nursing students (34). Ghrouz et al. (33) found that poor sleep quality was associated with anxiety in Indian university students (33). A recent systematic review summarized that sleep disturbances may aggravate the severity of anxiety symptoms, and thus, understanding comorbid anxiety and poor sleep may be important for the exploration of treatment strategies (35).

To the best of our knowledge, this is the first study to report a direct association of anxiety status, and anxiety level with inadequate sleep hygiene practices among young adults. However, thematically similar generalizations are entailed in some of the previous reports. Baroni et al. reported that an intervention called sleep course targeted to improve sleep hygiene practices also led to a decrease in anxiety levels in American college students (36). Similarly, Peltz et al. (37) using a moderator-mediator analysis found that (i) sleep hygiene was both directly and indirectly associated with anxiety levels in American adolescents whose school start time is before 8:30 am, and (ii) sleep hygiene was only directly associated with anxiety levels in American adolescents whose school start time was 8:30 am or later (37). A systematic review showed a constellation of poor sleep behaviors like inadequate sleep hygiene, and difficulty in initiating/maintaining sleep are common in children with anxiety disorders (38). Inadequate sleep hygiene practice is common in university students, due to continuous pressure to maintain a higher cumulative grade point average (cGPA), and personal socioeconomic position forcing some of them to work off-campus, all of these adds to their irregular sleeping schedules. Other evidence revealed that an increased co-sleeping tendency in anxious school-going children is found, with one in three children tending to co-sleep 2–4 times a week compared to non-anxious children (39). The use of portable electronic devices by children and adolescents before sleep has been proposed to cause displacement of time to sleep due to increased mental alertness, and psychological stimulation due to light exposure (40). To this end, inadequate sleep hygiene practice is common in university students, thus it is suggested that the universities should have appropriate advisory centers to provide specialized counseling tailored to improve sleep habits, and mental health.

Anxiety and increasing severity of anxiety were associated with psychological stress in the study population. Faravelli and Pallanti reported that distressing events in life lead to the development of anxiety symptoms (41). Perceived exam stress is common in university students with the fear of lower grades. Lower grades may consequently decrease prospects of finding jobs in the future, thoughts about this may increase the level of anxiety. Prolonged stress may impair the ability to perform well-during exams, and in the long-term makes an individual vulnerable to neuropsychiatric complications (42). Other aspects that may potentially increase stress in students are adjustments to the new campus life, fulfilling academic requirements, and simultaneous maintenance of social and academic life.

### Limitations of the Study

The study does not have an equal representation of females; nevertheless, the study satisfies the NIH mandate of inclusion of sex as a biological variable for reproducibility and replicability in science (43). Lesser female representation may also be related to the overall lower percentage of female students (about 30%) enrolled at MTU during the academic year. Similar studies involving Ethiopian university students also reported less participation from female students (16). It would be interesting to assess the relationship between courses of study and anxiety symptoms in future studies. Moreover, both regression models used in this study explained 20.4 and 24.1% of the variance. This is most plausibly explained by statistical consideration that some of the covariates associated with anxiety may have not been accounted for in the present study. This aspect should be further explored in future studies. Moreover, a clinical diagnosis of anxiety was not performed. However, it may not be out of place to mention that in the limited resource setting in which this study was performed, a comprehensive clinical neuropsychological assessment was not possible.

## Conclusion

The study found a high prevalence of anxiety and psychological anxiety levels in the participating Ethiopian university students. Both the status of anxiety and the increasing severity were associated with higher psychological stress, poor sleep, and inadequate sleep hygiene. Screening of these correlates of anxiety may help in the early identification and management of mental health.

## Data Availability Statement

The dataset generated during and/or analyzed during the current study is available in the Supplement File.

## Ethics Statement

The studies involving human participants were reviewed and approved by The Human Institutional Ethics Review Committee, College of Medicine and Health Sciences, Mizan-Tepi University, Mizan. The patients/participants provided their written informed consent to participate in this study.

## Author Contributions

MM, MS, JM, and BV conceptualized the study, methodology, and were involved in data collection and data curation. MM and MK did the data analysis and wrote and edited the manuscript. AHA, AA, SRP, and AB were involved in supervision. All authors contributed to the article and approved the submitted version.

## Conflict of Interest

SRP reports non-financial support from Somnogen Canada Inc. and occasional royalties for the editorial contribution from Springer during the conduct of the study and is an employee of Somnogen Canada Inc. The remaining authors declare that the research was conducted in the absence of any commercial or financial relationships that could be construed as a potential conflict of interest.

## Abbreviations

GAD: generalized anxiety disorder
PSS-10: perceived stress scale-10
SHI: sleep hygiene index
LSEQ-M: Leeds sleep evaluation questionnaire-Mizan
VAS: visual analog scale.
